# Antiviral Efficacy of Verdinexor *In Vivo* in Two Animal Models of Influenza A Virus Infection

**DOI:** 10.1371/journal.pone.0167221

**Published:** 2016-11-28

**Authors:** Olivia Perwitasari, Scott Johnson, Xiuzhen Yan, Emery Register, Jackelyn Crabtree, Jon Gabbard, Elizabeth Howerth, Sharon Shacham, Robert Carlson, Sharon Tamir, Ralph A. Tripp

**Affiliations:** 1 Department of Infectious Diseases, University of Georgia College of Veterinary Medicine, Athens, Georgia, United States of America; 2 Department of Pathology, University of Georgia College of Veterinary Medicine, Athens, Georgia, United States of America; 3 Karyopharm Therapeutics, Newton, Massachusetts, United States of America; The University of Chicago, UNITED STATES

## Abstract

Influenza A virus (IAV) causes seasonal epidemics of respiratory illness that can cause mild to severe illness and potentially death. Antiviral drugs are an important countermeasure against IAV; however, drug resistance has developed, thus new therapeutic approaches are being sought. Previously, we demonstrated the antiviral activity of a novel nuclear export inhibitor drug, verdinexor, to reduce influenza replication *in vitro* and pulmonary virus burden in mice. In this study, *in vivo* efficacy of verdinexor was further evaluated in two animal models or influenza virus infection, mice and ferrets. In mice, verdinexor was efficacious to limit virus shedding, reduce pulmonary pro-inflammatory cytokine expression, and moderate leukocyte infiltration into the bronchoalveolar space. Similarly, verdinexor-treated ferrets had reduced lung pathology, virus burden, and inflammatory cytokine expression in the nasal wash exudate. These findings support the anti-viral efficacy of verdinexor, and warrant its development as a novel antiviral therapeutic for influenza infection.

## Introduction

Influenza A viruses (IAVs) are emerging viruses that cause seasonal epidemics and periodic pandemics. Annual epidemics caused by IAVs are substantial causing >200,000 hospitalizations and >36,000 deaths in the United States each year, and worldwide, cause >20 million new cases of disease in children <5 years of age [1]. Despite substantial research on the mechanisms of action, only modest progress has been made in the development of IAV drugs. While annual influenza vaccines are available, antigenic drift and antigenic shift of emerging viruses naturally reduce vaccine efficacy and control. The current approved anti-influenza virus drugs are the M2 inhibitors (amantadine and rimantadine), and NA inhibitors (oseltamivir, zanamivir, peramivir) [2–4]. Drug-resistance to the M2-ion channel inhibitors is associated with single or multiple amino acid substitutions in the transmembrane region of M2, and more than 95% of transmissible, M2 inhibitor resistant influenza A virus strains carry the S31N mutation [5]. Further, both classes of approved anti-viral drugs are only effective if administered within 36–48 h of symptom onset having narrow therapeutic value [5]. Similarly, a number of mutations in the NA of viruses have been demonstrated by selection in the presence of NA inhibitors *in vitro*, thus new antivirals are actively being sought [6–10]. Host cellular genes required by the virus to replicate are appealing anti-viral targets due to their limited development of resistance/mutations, and because there are already small molecular drugs identified that target host genes which are currently under preclinical or clinical investigation and can potentially be repurposed.

Karyopharm Therapeutics has recently developed first-in-class, novel selective inhibitors of nuclear export (SINE) compounds using molecular modeling to screen a small virtual library of compounds against the NES groove of human exportin-1 (XPO1) [11, 12]. These compounds inhibit nuclear-cytoplasmic export by reversibly binding to the cargo recognition site of XPO1 and are orally bioavailable. SINE compounds were originally developed as therapeutics for various hematologic malignancies, as these drugs force the nuclear retention, accumulation, and functional activation of tumor suppressor proteins to limit oncogenesis [12–17]. One SINE compound, verdinexor (KPT-335), has completed a registration-directed study in companion dogs with newly diagnosed or first relapsed non-Hodgkin lymphoma (NHL). The FDA’s Center for Veterinary Medicine (CVM) considers the effectiveness and safety technical sections under the Minor Use Minor Species (MUMS) designation complete to support conditional approval for oral verdinexor as a single agent for the treatment of lymphoma in companion dogs under a New Animal Drug Application (NADA). Additionally, verdinexor was recently found to be effective to inhibit the replication of various influenza A and B virus strains by blocking the XPO1-mediated nuclear export of viral ribonucleoprotein complexes [18]. A preliminary *in vivo* study demonstrated verdinexor was efficacious in limiting virus burden and inflammatory cytokines expression in lungs of mice infected with a mouse-adapted strain of pandemic 2009 H1N1 influenza virus (A/California/04/09-MA), and mice that received oral verdinexor treatment also displayed reduced lung pathology and mortality upon lethal virus infection [18].

## Material and Methods

### Cell cultures and influenza virus stocks

Madin-Darby Canine Kidney (MDCK) cells (ATCC, CCL-34) and A549 cells (ATCC, CCL-185) were cultured in Dulbecco’s modified Eagle’s medium (DMEM), supplemented with 5% heat inactivated FBS (HyClone) in a 37°C incubator with 5% CO_2_. Parental and mouse-adapted influenza A/California/04/09, A/Philippines/2/82/X-79, and A/WSN/33 were propagated as previously described [18].

### *In vivo* mouse efficacy studies

BALB/c female mice (6–8 week-old) were obtained from the NCI (National Cancer Institute). All experiments and procedures were approved by the Institutional Animal Care and Use Committee of the University of Georgia. All experiments were performed with ten mice per group and repeated independently at least twice.

### Experimental procedures

Mice were intranasally (i.n.) inoculated with 0.1 mL of mouse-adapted A/California/04/09 or A/Philippines/2/82/X-79 at 10×MID_50_ (50%-mouse infectious dose; sub-lethal). Mice were treated by gavage *per os* (p.o.) with 20 mg/kg verdinexor at day 1 and 3 post-infection (pi) or 10 mg/kg oseltamivir twice daily after infection. A preliminary study was conducted to determine the most efficacious treatment regimen for verdinexor and the results showed that 20 mg/kg at day 1 and 3 was the most efficacious yet well tolerated (data not shown). At day 2 and 4 pi, mice were sacrificed and BAL fluids were collected to determine the amount of virus and pro-inflammatory cytokines secreted as described below. To determine the efficacy of verdinexor-oseltamivir combined treatment, mice were treated orally with 20 mg/kg verdinexor at day 2 and 4 pi, or in combination with 1 or 10 mg/kg oseltamivir twice daily on days 1 to 4. At day 5, mice were euthanized and lungs were collected for virus titration.

To determine the efficacy of late dosing of verdinexor, mice were infected with 10×MID_50_ of mouse-adapted A/California/04/09 and treated with 20 mg/kg verdinexor at day 1 and 3, day 2 and 4, day 3 and 5, day 4 and 5, or 10 mg/kg oseltamivir twice daily at day 1–4 pi. Lungs were harvested at day 6 pi for virus titration. For the survival study, mice were infected with 10×LD_50_ (50%-lethal dose) of mouse-adapted A/California/04/09 intranasally and treated with 5 or 10 mg/kg verdinexor at day 1 and 3 or 10 mg/kg oseltamivir twice daily for 4 days starting at day 1 pi. Mice were evaluated for weight, clinical signs, and survival for 14 days.

For pharmacokinetics (PK) study of oral verdinexor treatment, n = 3 male CD1 mice per time point (~28 g of weight) [BK Laboratory Animal Co. Ltd., Shanghai, China; qualification no. SCXK(SH) 2008–0016 12470] were used. Plasma verdinexor concentrations were determined by an ultrahigh-performance liquid chromatography-mass spectrometry method as previously described [18].

### Evaluation of virus titers

To evaluate the amount of virus shed, BAL supernatants were serially diluted and titered on MDCK cells for 72 h. Evaluation of virus titer in the lungs was performed as previously described [18]. Hemagglutination (HA) assays were performed using turkey RBCs and virus titers were calculated as 50% tissue culture infectious dose (TCID_50_) using the Spearman-Karber formula [19, 20].

### Bronchoalveolar lavage (BAL) for cytokines and cell type description

Levels of cytokines and chemokines in the BAL were analyzed using the MILLIPLEX MAP Mouse Cytokine/Chemokine Magnetic Bead Panel (EMD Millipore) and Luminex 200 system. For characterization of inflammatory cells infiltrates, BAL cells were resuspended in 100 μL of PBS, counted, and evaluated by flow cytometry. Briefly, cells were incubated with Fc Block, APC anti-mouse CD11c (HL3), PerCP-Cy5.5 anti-mouse Ly-6G/Ly-6C (Gr-1), PE anti-mouse CD49b/Pan-NK cells (DX5), and Alexa-488 anti-mouse CD3e (145-2C11) (BD Pharmingen) in staining buffer (1% BSA in PBS) for 60 min at 4°C. Cells were washed with staining buffer and fixed with 5% formaldehyde. All samples were run on a LSR-II flow cytometer (BD Biosciences) and analyzed using FlowJo (Tree Star).

### *In vivo* ferret efficacy studies

Young adult male ferrets (Triple F Farms) weighing between 800–1,000g (n = 3 per treatment group) were used for the study. Nasal washes were collected from influenza-naïve ferrets to establish basal cytokine expression profiles. Cells collected from nasal washes were concentrated by centrifugation and subjected to RNA isolation and ferret cytokines qRT-PCR as described below. Ferrets were i.n. inoculated with 0.1 mL of 10×ID_50_ (50%-infectious doses) A/California/04/09. Ferrets were treated orally with 15 or 20 mg/kg verdinexor daily (QD), or 10 mg/kg verdinexor or 5 mg/kg oseltamivir twice daily (BID) for 4 days starting at 1 h pre-infection. Ferrets were monitored for clinical symptoms (body temperature, activity, respiratory signs, and weight loss) daily. At day 2 and 4 pi, nasal washes were collected and centrifuged. Cells were counted and used to determine cytokines gene expression. At day 4 pi, ferrets were sacrificed and lungs were collected for histopathological analyses. A section of left cranial lung lobe was homogenized for use in determining lung virus titer as described. For PK analysis, plasma verdinexor concentrations were determined from n = 3 male ferrets.

### Quantitative RT-PCR analysis

Total RNA was isolated from nasal wash cells using the RNeasy Mini Kit (Qiagen) and cDNA were synthesized using the Verso cDNA Synthesis Kit and random hexamers as primer (Thermo Scientific). Quantitative PCR was performed using ferret *IFN-γ*, *IL-12p40*, *TNF-α*, and *GAPDH* gene specific primers [21] and RT^2^ SYBR Green qPCR Master Mix (SABioscience) in MX3005P thermocycler as previously described [18]. Ct values for cytokines were normalized to GAPDH and their expressions relative to basal samples were calculated using 2^(-ΔΔCt)^ formula.

### Histopathological analysis

The left cranial lung lobe was removed for virus titration. The remaining lungs were inflated with 10% buffered formalin then immersed in formalin. Following fixation, lung lobe were embedded in paraffin and four micron sections stained with hematoxylin and eosin (HE) as previously described [18]. Overall lung pathology was scored on a range of 0–4: 0 = unremarkable; 1 = minimal changes in bronchiolar epithelium with minimal perivascular inflammation; 2 = mild multifocal bronchial and/or bronchiolar epithelial changes with perivascular and peribronchial/bronchiolar inflammation; 3 = moderate, multifocal bronchial and/or bronchiolar epithelial changes with perivascular, peribronchial/bronchiolar and alveolar inflammation; and 4 = marked, diffuse bronchial and/or bronchiolar epithelial changes with perivascular, peribronchial/bronchiolar and alveolar inflammation.

### Statistical analyses

Statistical analyses were done using student’s t-test and results were presented as means ± standard errors (SE). Values of *p*≤0.05 were considered significant.

## Results and Discussion

### Verdinexor reduces influenza virus shedding, pro-inflammatory cytokines, and inflammatory cell infiltration

We have previously demonstrated the efficacy of verdinexor against IAV *in vitro* and *in vivo* in a mouse model of influenza infection [18]. Because verdinexor targets a host factor, it is expected that there will be limited to no development of resistance. As shown in S1 Fig, serial propagation of IAV in the presence of sub-virucidal dose of verdinexor did not result in a reduction of drug sensitivity *in vitro*. Thus, as part of further pre-clinical evaluation, and to expand on the initial *in vivo* findings, the *in vivo* efficacy of verdinexor was evaluated more comprehensively in mice, and in a second animal model of influenza pathogenesis, i.e. ferrets.

**Fig 1:**
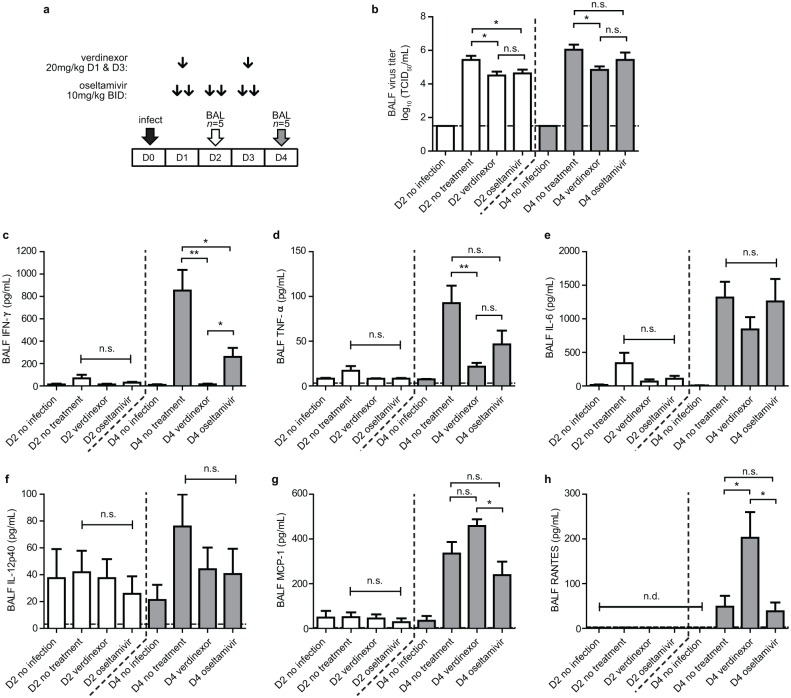
Verdinexor reduces influenza virus shedding and pro-inflammatory cytokines expression in BAL fluid. a) Experimental design: mice were infected with 10×MID_50_ of mouse-adapted A/California/04/09 intranasally and treated orally with 20 mg/kg verdinexor at day 1 and 3 or 10 mg/kg oseltamivir twice daily post-infection. Five mice per group were sacrificed at day 2 and 4 post-infection and BAL fluids were collected. b) Virus titers in the BAL were evaluated. Protein levels of c) IFN-γ, d) TNF-α, e) IL-6, f) IL-12p40, g) MCP-1, and h) RANTES in the BAL fluid were evaluated using the mouse cytokines/chemokines multiplex assay kit. *, *p*≤0.05; **, *p*≤0.01; n.s., not significant.

**Fig 2:**
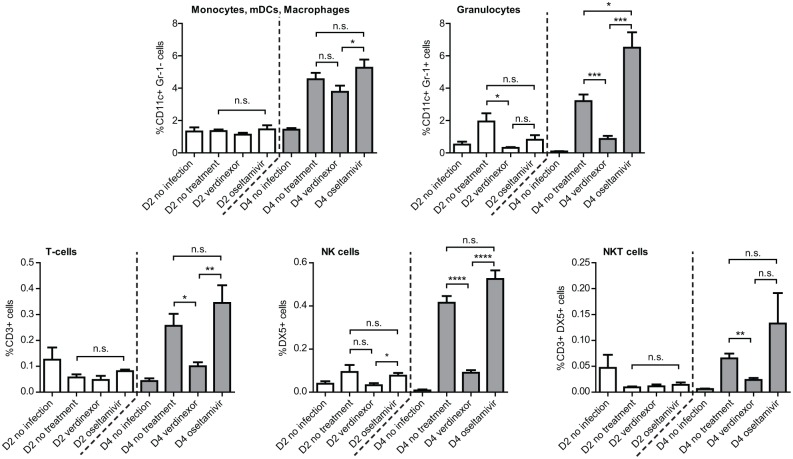
Verdinexor reduces inflammatory cell infiltration into the bronchoalveolar space in influenza infected mice. Mice were infected with 10×MID_50_ of mouse-adapted A/California/04/09 intranasally and treated orally with 20 mg/kg verdinexor at day 1 and 3 or 10 mg/kg oseltamivir twice daily post-infection. BAL cells from day 2 and 4 post-infection were characterized using flow cytometry. Percent of CD11c+Gr-1-, CD11c+Gr-1+, CD3+, DX5+, and CD3+DX5+ cells were plotted. The data are from five mice per experimental group per time point. *, *p*≤0.05; **, *p*≤0.01; ***, *p*≤0.001; ****, *p*≤0.0001.

In support of the finding that verdinexor reduces lung virus burden in influenza virus-infected mice, the amount of infectious virus shed was assessed at day 2 and 4 pi (Fig 1a and 1b). Mice that received 20 mg/kg verdinexor at day 1 and 3 pi had considerably (*p*≤0.05) reduced IAV in the bronchoalveolar lavaged fluid (BALF) at day 2 and 4 pi which was comparable to IAV observed in oseltamivir-treated mice. Since exacerbated inflammatory responses are associated with severe influenza virus infection, amount of pro-inflammatory cytokines and chemokines in the BALF were also assessed (Fig 1c–1h). Verdinexor-treated mice had significantly (*p*≤0.01) lower IFN-γ and TNF-α in the BALF compared to mock-treated mice. Levels of IL-6 and IL-12p40 were also reduced at day 4 pi, although these were not statistically significant. When compared to oseltamivir-treated mice, mice treated with verdinexor also showed significantly (*p*≤0.05) lower IFN-γ and slightly lower TNF-α at day 4 pi, although this difference was not statistically significant. Interestingly, levels of chemokines MCP-1 and RANTES were elevated in verdinexor-treated mice, although no significant increase in inflammatory cell recruitment was observed (Fig 2).

The type of BAL cells as determined by flow cytometry showed that verdinexor-treated mice had substantially lower immune cells infiltrates, in particular CD11c+Gr-1+ (granulocytes; *p*≤0.001), CD3+ (T cells; *p*≤0.05), DX5+ (NK cells; *p*≤0.0001), and CD3+DX5+ (NKT cells; *p*≤0.01) cells, but not CD11c+Gr-1- cells (dendritic cells and macrophages) (Fig 2). Representative flow cytometry plots for day 4 pi BAL samples are presented in S2 Fig. Together, these findings support the therapeutic use of verdinexor, and are consistent with the interpretation of the prior study regarding verdinexor prophylactic treatment to limit lung influenza virus replication and the pulmonary inflammatory response in infected mice [18].

### Efficacy of late-dosing with verdinexor

**Fig 3:**
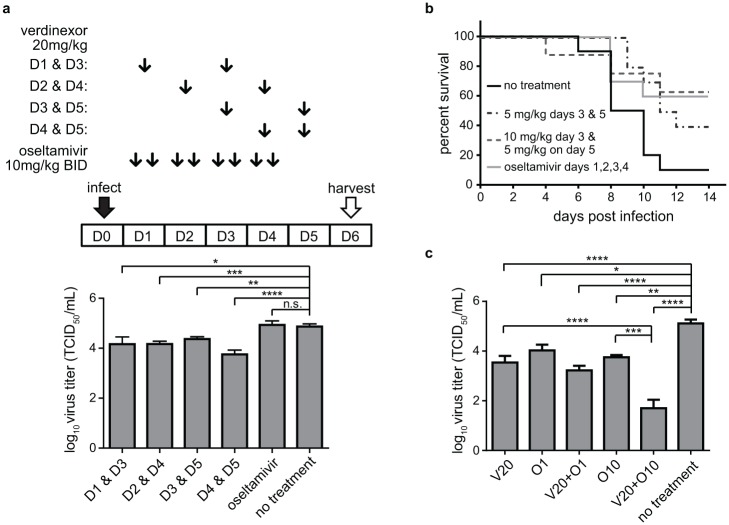
Verdinexor administration is efficacious when administered post-infection in mice. a) Mice were i.n. infected with 10×MID_50_ of mouse-adapted A/California/04/09 and orally treated with 20 mg/kg verdinexor at day 1 and 3, day 2 and 4, day 3 and 5, day 4 and 5, or 10 mg/kg oseltamivir twice daily at day 1–4 post-infection. Lungs were harvested at day 6 post-infection for virus titration. b) Mice were i.n. infected with 10×LD_50_ mouse-adapted A/California/04/09 and orally treated with 5 or 10 mg/kg verdinexor at day 1 and 3 or 10 mg/kg oseltamivir twice daily for 4 days starting at day 1 post-infection. Mice were evaluated for morbidity and mortality. Day of death was recorded either when a mouse was found deceased or when weight and health score required euthanasia. The data are from 8–10 mice per experimental group. c) Mice were infected with 10×MID_50_ of A/Philippines/2/82/X-79 (H3N2). Mice were treated orally with 20 mg/kg verdinexor (V20) on both day 2 and 4 following infection, alone or in combination with 1 (O1) or 10 mg/kg (O10) oseltamivir twice daily on days 1 to 4. At day 5, mice were euthanized and lungs were collected for virus titration. The data are from 8–12 mice per experimental group. *, *p*≤0.05; **, *p*≤0.01; ***, *p*≤0.001; ****, *p*≤0.0001.

Ensuing studies were designed to evaluate the antiviral effect of a late dosing regimen with verdinexor. A group of mice infected with influenza A/California/04/09-MA were treated with 20 mg/kg verdinexor every-other-day (QOD) for a total of 2 doses, starting at day 1 pi. Additional groups of mice received similar but a delayed regimen starting at day 2, 3, or 4 pi. Lung virus titers were evaluated at day 6 pi (Fig 3a). Delayed verdinexor treatment, i.e. administered at day 2 pi or later, was able to reduce lung virus titer to comparable if not better levels than the regimen started at day 1 pi (*p*≤0.05). The most significant (*p*≤0.001) reduction in lung virus titer was observed when one of the doses was given at day 4 pi which is the peak of virus replication in the absence of treatment.

The efficacy of late verdinexor treatment was assessed for improving survival following lethal influenza virus challenge (Fig 3b). When verdinexor treatment was delayed, mortality was still reduced in groups that received 5 mg/kg verdinexor at day 3 and 5, or 10 mg/kg at day 3 and 5 mg/kg at day 5 pi (*p*≤0.05) compared to non-treated mice. No significant difference in survival was observed between groups that received delayed verdinexor treatment and a control group receiving standard twice-daily oseltamivir regimen initiated immediately following infection. The results demonstrate that verdinexor is efficacious even when given at later time points post-infection.

### Efficacy of verdinexor-oseltamivir combination treatment

The efficacy of verdinexor given alone or in combination with oseltamivir against another IAV subtype was evaluated (Fig 3c). Mice were infected with influenza A/Philippines/2/82/X-79 (H3N2) and either not treated, treated with 1 or 10 mg/kg oseltamivir only, 20 mg/kg verdinexor only, or treated with verdinexor and oseltamivir in combination. On day 5 pi, mice were euthanized and lungs were collected to determine virus titers. All treatment groups had appreciably lower lung virus titers compared to the non-treated group. Importantly, mice treated with a combinational therapy of 20 mg/kg verdinexor and 10 mg/kg oseltamivir had significantly lower titers when compared to those that received 20 mg/kg verdinexor only (*p*≤0.0001) or 10 mg/kg oseltamivir only (*p*≤0.001), with 4 of the 10 mice had levels of virus at or below the assay detection limit. This result further demonstrates the efficacy of verdinexor against multiple IAV subtypes, and that it augments the antiviral effect of oseltamivir.

### Verdinexor limits IAV replication, lung pathology, and inflammation in ferrets

**Fig 4:**
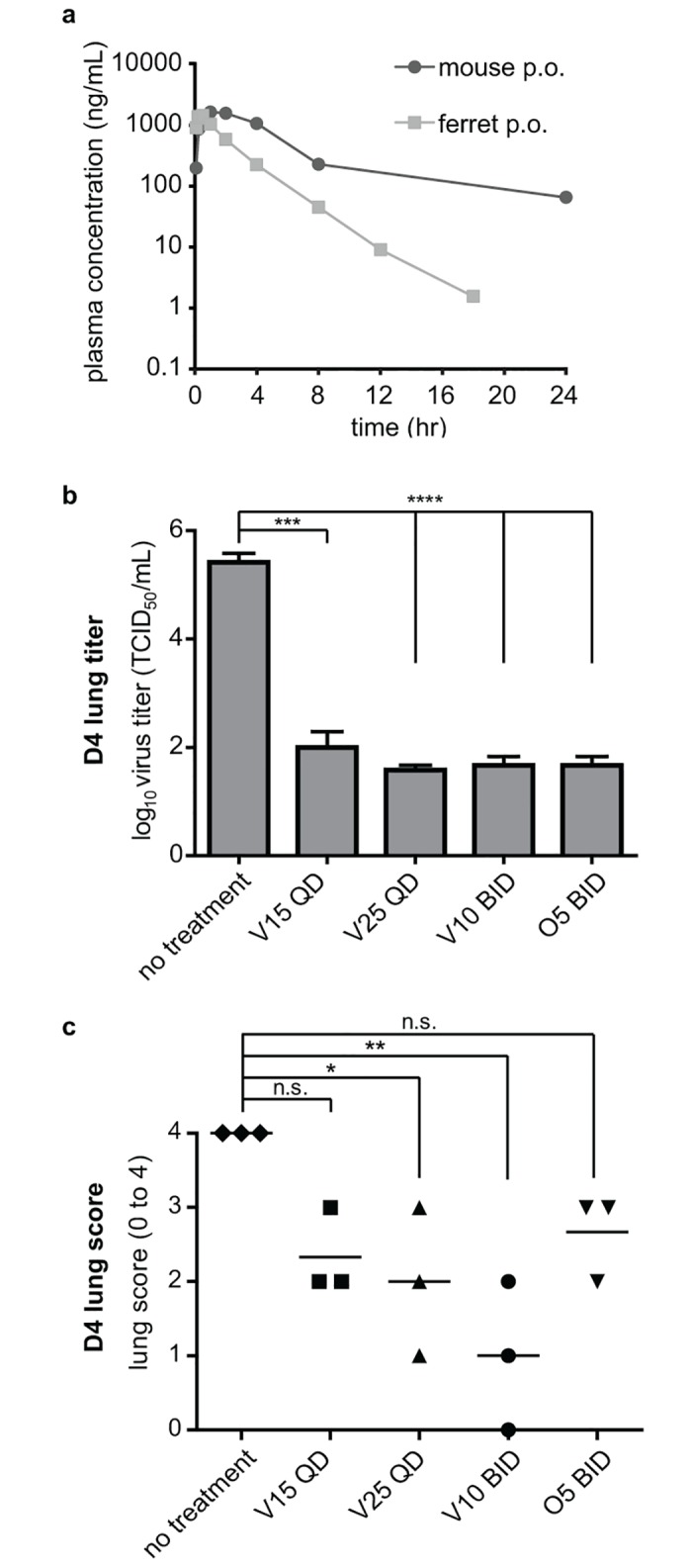
Verdinexor limits influenza and lung pathology in ferrets. a) Verdinexor plasma concentrations were determined in ferrets that received 5 mg/kg verdinexor orally. Verdinexor’s pharmacokinetic profile in mice that received 10 mg/kg verdinexor p.o. was previously published [18]. b) Ferrets were i.n. infected with 10×ID_50_ A/California/04/09 and orally treated with 15 (V15) or 25 mg/kg (V25) verdinexor daily (QD), or 10 mg/kg verdinexor (V10) or 5 mg/kg oseltamivir (O5) twice daily (BID) for 4 days starting at 1 h pre-infection. Lungs were harvested at day 4. Sections from left cranial lobes were homogenized to evaluate lung virus titers. The remaining lungs were fixed, sectioned, and subjected to HE staining for histopathological analysis. c) Lungs were scored based on observed pathology. The data are from three ferrets per experimental group. *, *p*≤0.05; **, *p*≤0.01; ***, *p*≤0.001, ****, *p*≤0.0001.

The mouse model has been used extensively to study the host response to IAV primarily because of lower costs, reagent availability, and precedence [22]. However, mice do not accurately emulate the clinical signs of IAV in humans, in contrast to the ferret model, which more closely emulates IAV disease pathogenesis [22, 23]. Furthermore, as in human, predominance of α2,6-linkage sialic acid (SA) is found in the upper respiratory track of ferrets, whereas α2,3 SA are more predominant in mice [23]. A pharmacokinetic (PK) study of oral verdinexor administration in ferrets was conducted to determine the feasibility of investigating the *in vivo* antiviral efficacy in ferrets (Fig 4a and Table 1). The study showed that verdinexor is orally bioavailable in ferrets, but was more rapidly cleared from the plasma compared to mice [18]. To address this, ferrets were dosed daily (QD) or twice daily (BID) for four days for a total of 4 or 8 doses, respectively, starting at 1 h prior to infection with A/California/04/09. Nasal washes were collected from infected ferrets at day 2 and 4 pi for cytology and evaluation of cytokines expression. Ferrets were sacrificed at day 4 pi and lungs were collected for evaluation of virus titers and pathology. The ferrets that received verdinexor at 15 mg/kg QD, 25 mg/kg QD, 10 mg/kg BID, or the standard oseltamivir treatment regimen showed considerably (*p*≤0.001) reduced lung virus titers (Fig 4b). Histopathology of the lungs revealed significant amelioration of influenza-associated lung pathology and inflammation in ferrets that received verdinexor at 25 mg/kg QD (*p*≤0.05) or 10 mg/kg BID (*p*≤0.01) (Fig 4c). However, limited reduction in pathology was observed in ferrets that received lower verdinexor dose of 15 mg/kg QD or oseltamivir. Representative histopathology slides and detailed pathology report are included in S3 Fig.

**Table 1:** Pharmacokinetic parameters of verdinexor following a *per os* dose of 5 mg/kg in male ferrets.

PK parameters	Unit	Mean	SD	CV
T_max_	h	0.361	0.241	66.7
C_max_	ng/mL	1494	1053	70.5
Terminal T_1/2_	h	1.84	0.148	8.08
AUC_last_	h*ng/mL	3506	1850	52.8
AUC_INF_	h*ng/mL	3513	1845	52.5

**Fig 5:**
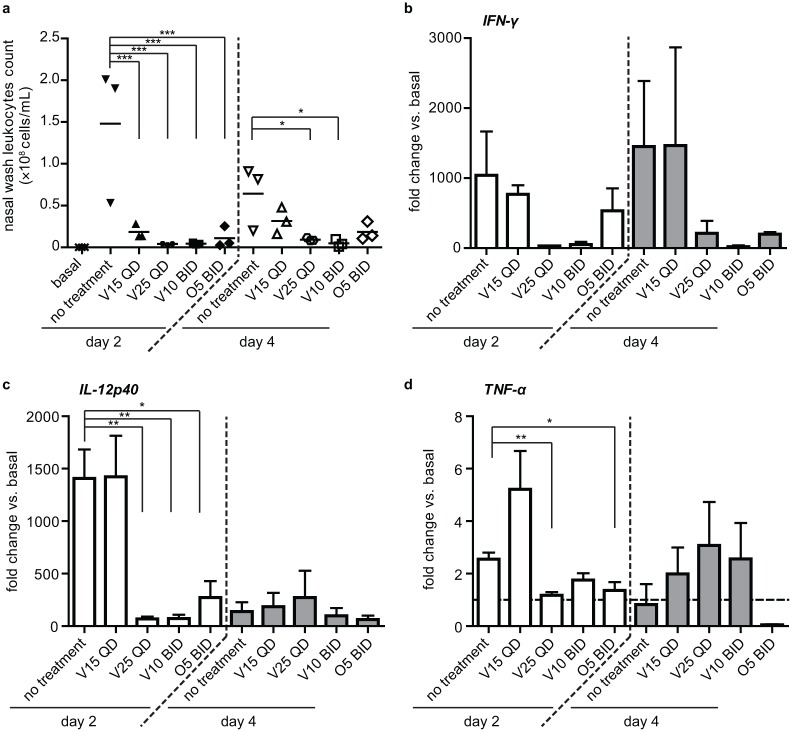
Verdinexor treatment reduces immune cell infiltration and pro-inflammatory cytokine expression and in influenza-infected ferrets. Ferrets were i.n. infected with 10×ID_50_ A/California/04/09 and orally treated with 15 (V15) or 25 mg/kg (V25) verdinexor daily (QD), or 10 mg/kg verdinexor (V10) or 5 mg/kg oseltamivir (O5) twice daily (BID) for 4 days starting at 1 h pre-infection. Ferrets nasal washes were collected pre-treatment/infection (basal) and at day 2 and 4 post-infection. a) Number of leukocytes from nasal washes was counted. Total RNA was isolated from nasal wash cells and the expression of b) *IFN-γ*, c) *IL-12p40*, and d) *TNF-α* genes relative to that of *GAPDH* was evaluated by qRT-PCR. The data are from three ferrets per experimental group. *, *p*≤0.05; **, *p*≤0.01; ***, *p*≤0.001.

To understand the effect of verdinexor on inflammatory responses associated with IAV, bronchoalveolar immune cell infiltration and cytokines expression was determined in ferret nasal washes. Lower numbers of infiltrating leukocytes were observed for all ferrets that received verdinexor or oseltamivir treatment at day 2 pi (*p*≤0.001), while only ferrets receiving verdinexor at 25 mg/kg QD or 10 mg/kg BID showed significant (*p*≤0.05) reduction at day 4 pi (Fig 5a).

Due to limited antibodies available for ferret cytokines detection, qRT-PCR methods were employed to detect cytokines gene expression in cells harvested from the nasal washes (Fig 5b–5d). Ferrets that received verdinexor at 25 mg/kg QD (*p*≤0.01), 10 mg/kg BID (*p*≤0.01), or oseltamivir (*p*≤0.05) showed reduced *IL-12p40* expression in nasal exudates collected at day 2 pi. Ferrets that received 25 mg/kg QD verdinexor (*p*≤0.01) or oseltamivir (*p*≤0.05) also showed reduced *TNF-α* expression at day 2 pi. Except for the ferrets that received the lowest dose of verdinexor (15 mg/kg QD), the other treated ferrets also displayed a trend toward reduced *IFN-γ* expression at day 2 and 4 pi, although these reductions were not statistically significant. These findings demonstrate verdinexor efficacy to limit influenza virus infection, and the associated pathology and inflammation in a second animal model of influenza infection, i.e. the ferret.

## Conclusions

Together, these studies show that verdinexor is a potent inhibitor of IAV replication *in vivo*, which was shown in two animal models of IAV using the pandemic 2009 influenza virus strain. Furthermore, virus cultivation in the presence of sub-efficacious dose of verdinexor did not appear to result in the emergence of drug resistance. In this study, the *in vivo* late dosing result expands on the previously published findings on verdinexors antiviral efficacy in the mouse model [18]. As oseltamivir has to be administered within 48 hours of infection for its optimal effect [5], this result also demonstrates the added advantage of verdinexor treatment when given late following infection. The current study also shows the multifaceted ability of verdinexor to ameliorate influenza-associated diseases, in which reductions of both lung virus titer and inflammation were observed in verdinexor-treated mice and ferrets. It is expected that these studies will serve as important stepping stones for future clinical evaluations of verdinexor for its anti-influenza indication in human. Further, other human pathogenic viruses have also been reported to co-opt the host’s XPO1 pathway for nuclear exit, including the herpes simplex virus type-1 virus (HSV-1), human cytomegalovirus (HCMV), and the severe acute respiratory syndrome coronavirus (SARS-CoV) [24–26]. Thus, it is likely that verdinexor can also potentially inhibit replication of these viruses serving as a broad-spectrum antiviral.

Verdinexor has been tested in a phase I clinical trial in healthy volunteers and was found to be safe and well-tolerated (ClinicalTrials.gov NCT02431364). Furthermore, a closely related SINE compound, selinexor (KPT-330) is currently undergoing multiple separate phase I and II studies in human patients with advanced, relapsed, and refractory cancers (such as ClinicalTrials.gov NCT01607905 and NCT01607892). Preliminary results from these studies demonstrated that selinexor is generally well tolerated, with good oral exposure and evidence of anti-cancer activity [27–29], a finding which would also advocate for verdinexor’s safety profile for its use in humans.

## Supporting Information

S1 Fig Assessment of *in vitro* drug resistance. Influenza A/WSN/33 was serially passaged in A549 cells in the absence or presence of sub-virucidal dose of verdinexor and the sensitivity of the passaged virus to verdinexor was determined. a) Outline of the experimental procedure. For serial propagation of the virus, A549 cells were left untreated (b) or treated with 0.2 μM verdinexor for 2 h (c), then infected with influenza A/WSN/33 at MOI = 0.001. At 72 hpi, supernatants containing virus were collected. An aliquot of the supernatant was taken and diluted 1:100 to infect a new plate of A549 cells, while the rest was kept at -80°C for the assessment of drug resistance. The virus was serially passaged 10 times in quadruplicate wells. At the conclusion of serial passaging experiment, passaged virus aliquots were thawed and titered by plaque assay. To assess potential drug resistance that may arise from serial passaging in sub-virucidal dose of verdinexor, stock virus (WT), passage-2 (P2), -4 (P4), -6 (P6), -8 (P8), and -10 (P10) viruses were used to infect A549 cells that were pre-treated with DMSO (-; black bars) or with a virucidal dose of verdinexor (5 μM) (+; white bars) for 2 h at MOI = 0.01. At 48 hpi, viral titer in the supernatant was evaluated using the TCID_50_ method (mean virus titer ± SEM). Virus cultured in the presence of sub-virucidal dose of verdinexor remained sensitive to 5 μM verdinexor, even after 10 passages (white bars in panel c). There was also an apparent overall reduction of viral growth and/or fitness in virus passaged in the presence of verdinexor (black bars in panel c).
(TIF)

S2 Fig Verdinexor reduces inflammatory cell infiltration into the bronchoalveolar in IAV mice. BAL cells from day 2 and 4 post-infection were characterized using flow cytometry. Representative FACS plots from day 4 BAL cells were shown. The data are from five mice per experimental group per time point.
(EPS)

S3 Fig Representative HE slides from lungs of ferrets infected with influenza A/California/04/09. a) Normal ferret lung: normal bronchiole (B), thin alveolar septa, and empty alveoli were observed. b) Lung of non-treated, influenza-infected ferret: bronchioles were not readily identified due to necrosis of the wall and obliteration of the lumen by inflammation which spilled out into and filled alveoli. Moderate peribronchiolar and perivascular infiltration of mostly lymphocytes was present. This animal had a lung score of 4. c) Lung of infected ferret treated with 15 mg/kg QD Verdinexor: bronchiolar epithelium was partially necrotic and neutrophils and macrophages partially filled the lumen (B). Mild peribronchiolar and perivascular inflammation was present. Alveolar collapse and mild inflammation made the parenchyma appear consolidated (A). This animal had a lung score of 2. D. d) Lung of infected ferret treated with 25 mg/kg QD Verdinexor: the bronchiolar epithelium was mostly necrotic and the lumen filled with neutrophils and macrophages (B) that were also found in the surrounding alveoli. Moderate peribronchiolar and perivascular inflammation of mostly lymphocytes was present. Alveolar septa were slightly thickened. This animal had a lung score of 2. e) Lung of infected ferret treated with 10 mg/kg BID Verdinexor: the bronchiole (B) was lined by hyperplastic epithelium and there was mild peribronchiolar and perivascular infiltration of mostly lymphocytes. Alveolar involvement was not present. This animal had a lung score of 2. f) Lung of Oseltamivir-treated infected ferret: a bronchiole was lined by hyperplastic epithelium and the lumen filled with neutrophils and macrophages admixed with lymphocytes (B) which spilled out into surrounding alveoli. There was mild peribronchiolar and perivascular inflammation of mostly lymphocytes. Alveolar septa were thickened by mild type-II cell hyperplasia and a small number of neutrophils and mononuclear cells were present in the alveoli (A). This animal had a lung score of 3. Bar = 200μm.
(TIF)
