# Supplementary figures and images for: Antiviral Efficacy of Verdinexor In Vivo in Two Animal Models of Influenza A Virus Infection
Source: PLoS One. 2016 Nov 28;11(11):e0167221. doi: 10.1371/journal.pone.0167221 (PMC5125695; doi:10.1371/journal.pone.0167221)

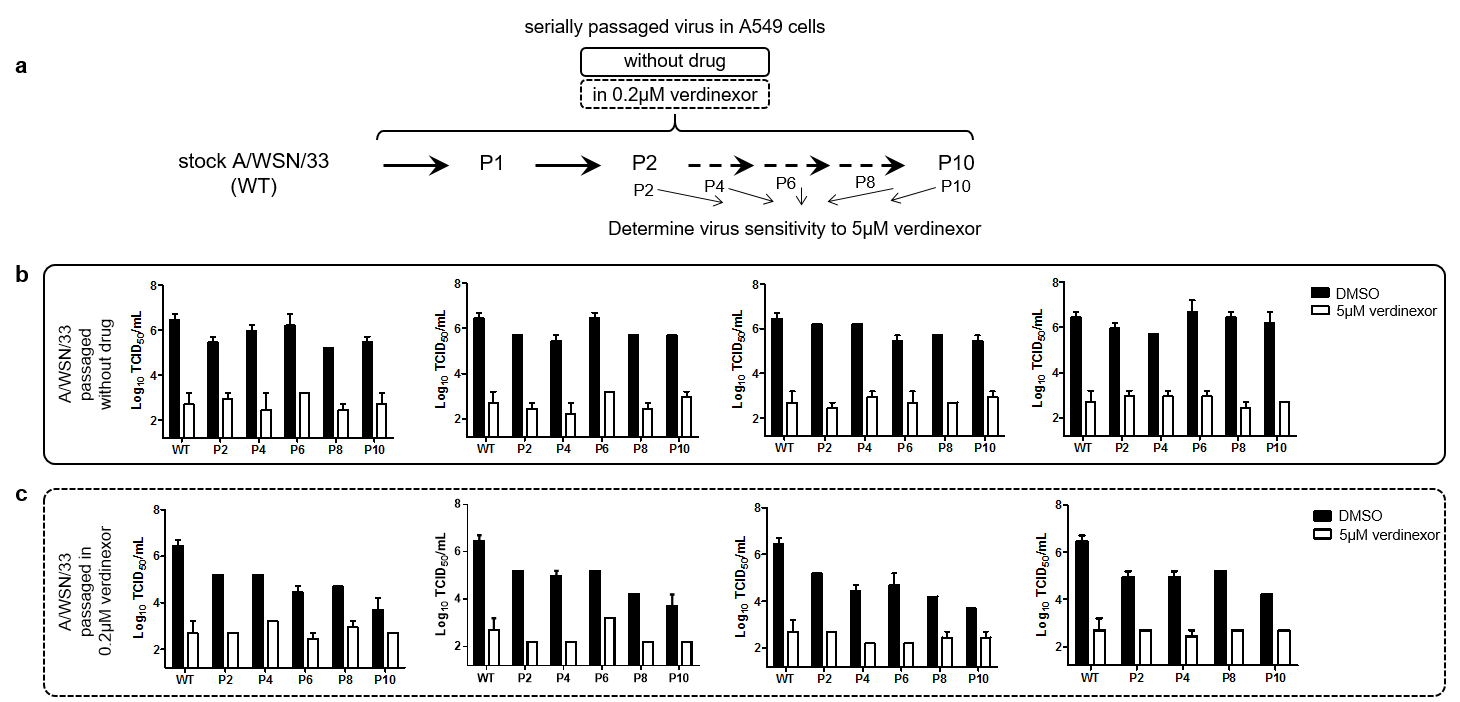

Supplement: S1 Fig — Influenza A/WSN/33 was serially passaged in A549 cells in the absence or presence of sub-virucidal dose of verdinexor and the sensitivity of the passaged virus to verdinexor was determined. a) Outline of the experimental procedure. For serial propagation of the virus, A549 cells were left untreated (b) or treated with 0.2 μM verdinexor for 2 h (c), then infected with influenza A/WSN/33 at MOI = 0.001. At 72 hpi, supernatants containing virus were collected. An aliquot of the supernatant was taken and diluted 1:100 to infect a new plate of A549 cells, while the rest was kept at -80°C for the assessment of drug resistance. The virus was serially passaged 10 times in quadruplicate wells. At the conclusion of serial passaging experiment, passaged virus aliquots were thawed and titered by plaque assay. To assess potential drug resistance that may arise from serial passaging in sub-virucidal dose of verdinexor, stock virus (WT), passage-2 (P2), -4 (P4), -6 (P6), -8 (P8), and -10 (P10) viruses were used to infect A549 cells that were pre-treated with DMSO (-; black bars) or with a virucidal dose of verdinexor (5 μM) (+; white bars) for 2 h at MOI = 0.01. At 48 hpi, viral titer in the supernatant was evaluated using the TCID50 method (mean virus titer ± SEM). Virus cultured in the presence of sub-virucidal dose of verdinexor remained sensitive to 5 μM verdinexor, even after 10 passages (white bars in panel c). There was also an apparent overall reduction of viral growth and/or fitness in virus passaged in the presence of verdinexor (black bars in panel c). (TIF) [file pone.0167221.s001.tif]

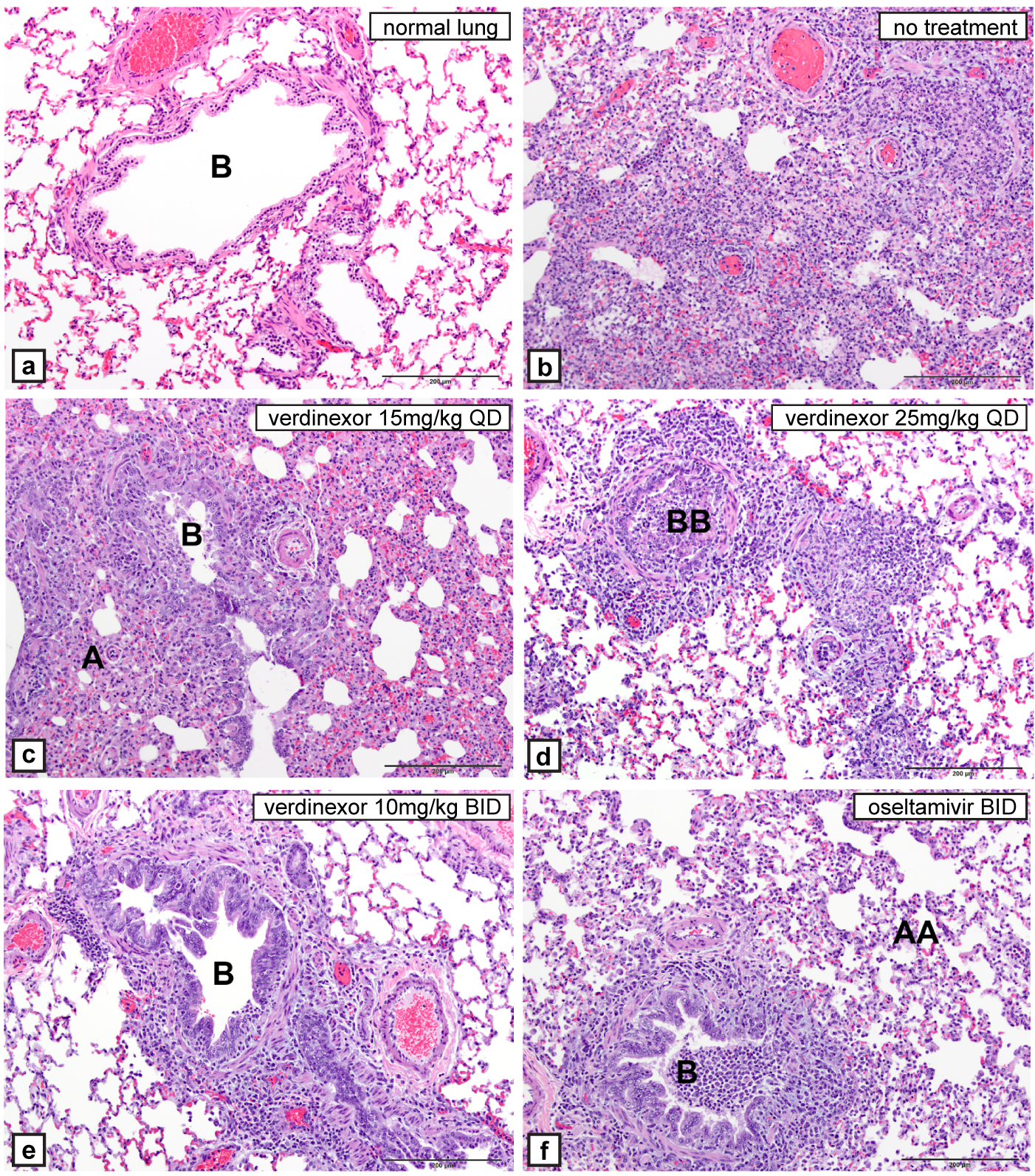

Supplement: S3 Fig — a) Normal ferret lung: normal bronchiole (B), thin alveolar septa, and empty alveoli were observed. b) Lung of non-treated, influenza-infected ferret: bronchioles were not readily identified due to necrosis of the wall and obliteration of the lumen by inflammation which spilled out into and filled alveoli. Moderate peribronchiolar and perivascular infiltration of mostly lymphocytes was present. This animal had a lung score of 4. c) Lung of infected ferret treated with 15 mg/kg QD Verdinexor: bronchiolar epithelium was partially necrotic and neutrophils and macrophages partially filled the lumen (B). Mild peribronchiolar and perivascular inflammation was present. Alveolar collapse and mild inflammation made the parenchyma appear consolidated (A). This animal had a lung score of 2. D. d) Lung of infected ferret treated with 25 mg/kg QD Verdinexor: the bronchiolar epithelium was mostly necrotic and the lumen filled with neutrophils and macrophages (B) that were also found in the surrounding alveoli. Moderate peribronchiolar and perivascular inflammation of mostly lymphocytes was present. Alveolar septa were slightly thickened. This animal had a lung score of 2. e) Lung of infected ferret treated with 10 mg/kg BID Verdinexor: the bronchiole (B) was lined by hyperplastic epithelium and there was mild peribronchiolar and perivascular infiltration of mostly lymphocytes. Alveolar involvement was not present. This animal had a lung score of 2. f) Lung of Oseltamivir-treated infected ferret: a bronchiole was lined by hyperplastic epithelium and the lumen filled with neutrophils and macrophages admixed with lymphocytes (B) which spilled out into surrounding alveoli. There was mild peribronchiolar and perivascular inflammation of mostly lymphocytes. Alveolar septa were thickened by mild type-II cell hyperplasia and a small number of neutrophils and mononuclear cells were present in the alveoli (A). This animal had a lung score of 3. Bar = 200μm. (TIF) [file pone.0167221.s003.tif]
